# Selenium-derivatized Nucleic Acids for Phasing, Crystal Growth, Structure Determination and Beyond

**DOI:** 10.1063/4.0000802

**Published:** 2025-10-27

**Authors:** Zhen Huang, Hehua Liu, Jianhua Gan

**Affiliations:** 1 SeNA Research Institute, College of Life Sciences, Hubei University, Wuhan, Hubei, China; 2 School of Life Sciences, Fudan University, Shanghai, Shanghai, China

## Abstract

Nucleic acids play important roles in life processes, such as genetic material, gene regulation, transcription, protein synthesis, signaling, catalysis, and viral infection. Nucleic acids are considered as ones of the keys to understand the cellular functions and disease mechanisms. Nucleic acid structure biology, especially DNA and RNA X-ray crystallography, can greatly accelerate the studies of nucleic acid-protein structures, functions and mechanisms. However, due to the challenges in crystallization, heavy-atom derivatization and phase determination, X-ray crystallography of nucleic acids (DNA and RNA, and their protein complexes) is challenging, and the structures of many nucleic acids and their protein complexes haven’t been resolved. To address these challenges, we have developed the selenium-atom specifically derivatized nucleic acids (SeNA), which can not only enhance the derivatization and diffraction phase determination, but also facilitate crystal growth without significant structure perturbation. This method has huge advantages over the traditional methods, such halogen derivatizations, heavy-metal socking, and molecular replacement. Recently, our laboratory has been further exploring Se-nucleic acid (a novel paradigm of nucleic acids), probing nucleic acid molecules and their protein complexes at the atomic level, and expanding its potential applications in structure-and-function investigations, gene re-design, bio-informatics, molecular diagnostics and macromolecular drugs, especially mRNA therapeutics, antisense, siRNA and other potential nucleic acid drugs.

